# A New Strategy to Prevent Emerging *Lactococcus garvieae* Infections by Using Organic Acids as Antimicrobials In Vitro and Ex Vivo

**DOI:** 10.3390/ijms26073423

**Published:** 2025-04-05

**Authors:** Igori Balta, Florin Dan Simiz, Ducu Stef, Ioan Pet, Gabi Dumitrescu, Tiberiu Iancu, Iuliana Cretescu, Nicolae Corcionivoschi, Lavinia Stef

**Affiliations:** 1 Faculty of Bioengineering of Animal Resources, University of Life Sciences King Mihai I of Romania from Timisoara, 300645 Timisoara, Romania; balta.igori@usvt.ro (I.B.); ioanpet@usvt.ro (I.P.); gabidumitrescu@usvt.ro (G.D.); 2 Faculty of Veterinary Medicine, University of Life Sciences King Mihai I of Romania from Timisoara, 300645 Timisoara, Romania; 3 Faculty of Food Engineering, University of Life Sciences King Mihai I of Romania from Timisoara, 300645 Timisoara, Romania; ducustef@usab-tm.ro; 4 Faculty of Management and Rural Development, University of Life Sciences King Mihai I of Romania from Timisoara, 300645 Timisoara, Romania; tiberiuiancu@usvt.ro; 5 Department of Functional Sciences, Faculty of Medicine, Victor Babes University of Medicine and Pharmacy, 300041 Timisoara, Romania; iuliana.cretescu@umft.ro; 6 Bacteriology Branch, Veterinary Sciences Division, Agri-Food and Biosciences Institute, Belfast BT4 3SD, Northern Ireland, UK; nicolae.corcionivoschi@afbini.gov.uk; 7 Academy of Romanian Scientists, 050044 Bucharest, Romania

**Keywords:** *Lactococcus garvieae*, organic acids, virulence, haemolysis, proinflammatory cytokines, natural antimicrobials

## Abstract

The ever-growing global demand for animal protein forces the aquaculture industry to expand at a pace which imposes significant challenges in maintaining sustainable practices. This study aimed to investigate the efficacy of an organic acid mixture (Aq) in mitigating *Lactococcus garvieae* (*L. garvieae*) virulence through its effects on bacterial virulence (EPS production, biofilm, and haemolytic factors) and host pathogenicity, including its adherence to CHSE-214 cells, haemolysis, and proinflammatory responses. Our findings reveal that Aq significantly inhibits *L. garvieae* growth at a 0.125% concentration, suppresses EPS-related gene expression, reduces biofilm formation, and reduces cytotoxicity in fish epithelial cells (CHSE-214). Moreover, Aq decreased haemolysing gene expression (*hly1*, *hly2* and *hly3*) and attenuated red blood cell haemolysis, a hallmark of *L. garvieae* pathogenicity. Lastly, Aq was demonstrated to induce modulation in the host immune responses, lowering IL-1β and IL-8 expression, which are critical mediators of inflammation and pathogen recruitment. Therefore, we conclude that the main mechanism of action of Aq involves inhibiting *L. garvieae* adhesion to epithelial cells, reducing EPS production, and downregulating key virulence-associated genes (e.g., *hly1*, *hly2*, and *hly3*). After preventing *L. garvieae* adherence and suppressing proinflammatory cytokine expression (IL-1β and IL-8), Aq disrupts the pathogen’s ability to breach epithelial barriers and induce red blood cell lysis, thereby mitigating its virulence and pathogenicity. Our results emphasised the potential of Aq as an alternative non-antibiotic intervention for controlling piscine lactococcosis, advancing our understanding of *L. garvieae* pathogenesis and providing the foundation for the future integration of environmentally friendly antimicrobials into aquaculture disease management.

## 1. Introduction

*Lactococcus garvieae* (*L. garvieae*), a Gram-positive lactic acid bacterium belonging to the *Streptococcaceae* family, has gained notoriety as a significant pathogen in aquaculture, particularly in freshwater and marine environments—causing piscine lactococcosis [1,2,3,4]. Initially described in 1983, it was first isolated from cases of bovine mastitis and later recognised as a causative agent of fish diseases [4]. Historical records trace its emergence as a fish pathogen back to Japan in the 1970s, initially comprehended as *Enterococcus seriolicida*, where it was implicated in severe outbreaks of lactococcosis in yellowtail (*Seriola quinqueradiata*), which marked the beginning of its recognition as a global threat to aquaculture [5,6,7,8]. The pathogen’s rapid spread through Europe became evident in the early 1990s, with the first documented cases in Spain in rainbow trout (*Oncorhynchus mykiss*) [1,4]. Over the following decades, infections were reported across the Mediterranean, the Balkans, and beyond, affecting both cold- and warm-water fish species [4]. The pathogen spread was further reported near the enclosed regions in the Middle East, Australia, Canada, Asia, and the Americas, accentuating *L*. *garvieae*’s unique adaptability to various aquatic ecosystems and its role as a persistent challenge to fish health worldwide [1,4,9].

The Food and Agriculture Organisation (FAO) estimated that the global demand for animal protein will rise substantially—from an estimated 20 kg per capita to 40 kg per day by 2030 [10,11]. The surge in protein consumption has pushed production systems for aquaculture to the limit and has driven an increase in demand for fish meal and fish oil—two critical but finite components in aquafeed formulations—to parallel levels [10]. Simultaneously, aquaculture’s rapid growth as a global industry brings challenges, particularly bacterial disease management affecting production [11]. High-value salmon species like rainbow trout (Oncorhynchus mykiss), widely farmed in Europe, Asia, and the U.S., face threats from *L. garvieae* [1,4,12]. The economic impact of *L. garvieae* outbreaks goes beyond direct fish stock losses [12]. Recent studies from European trout farms indicated challenges with a lowered feed efficiency, impaired performance, and marked losses are attributed to mortality-related expenses, including the costs associated with waste disposal and elevated medication costs due to the pathogen’s persistence in aquaculture environments, aided by biofilm formation and survival in reservoirs, further complicating eradication efforts [1,4]. In regions like Iran, Turkey, France, and Italy—key contributors to global trout production—the pathogen’s presence threatens local economies and global protein supply chains [4,13]. Fish farming is a vital sector providing food security, generating billions in annual revenue, supplying <5 billion people and ≈600 million livelihoods worldwide, which makes the control of lactococcosis and other diseases a prominent issue [1].

Affected fish typically exhibit systemic and localised symptoms indicative of extensive vascular damage and organ dysfunction [4,14]. The clinical signs of lactococcosis include anorexia, darkened skin (melanosis), and erratic swimming behaviours, often progressing to spiral or lethargic swimming [1,14,15,16]. External symptoms frequently observed are exophthalmia, haemorrhages in the periorbital area, base of fins, and perianal regions, as well as swollen abdomens and anal prolapses [1,4,15,16,17]. Internally, infected fish display gross pathological changes such as the congestion and enlargement of internal organs, yellowish exudate on the brain surface, necrotic lesions in the liver and spleen, and the accumulation of turbid ascitic fluid in the abdominal cavity [1,16]. These signs reflect the systemic and often overwhelming impact of *L*. *garvieae* on fish physiology.

Considering the growing trend for sustainable aquaculture practices, the inclusion of natural antimicrobials into management strategies could represent a viable path forward for managing lactococcosis and other diseases provoked by different pathogens. Their ability to reduce morbidity and mortality while minimising ecological and health risks positions them as a centrepiece in the development of holistic approaches to disease management in aquaculture. Medicinal plant derivatives, probiotics, organic acids, and bioactive substances are promising due to their multifunctional in vitro and ex vivo properties in different marine models [18,19,20,21]. Phytogenic supplements and mixtures of organic acids and plant extracts contain bioactive compounds such as polyphenols and terpenoids, having previously demonstrated antioxidant, anti-inflammatory, and immunomodulatory activities to enhance fish health against bacterial infections, and promote growth and improve feed efficiency, benefiting disease management in fish [14,18,19,20,21,22,23]. Additionally, bee-derived substances like propolis and honey exhibit antimicrobial properties against pathogens like *L*. *garvieae* [23]. These natural alternatives are environmentally friendly, safe, and residue-free in aquaculture and could in aid disease prevention by modulating fish gut microbiomes, outcompeting pathogens, and enhancing health without invasiveness or residues. Prophylactic measures, including the routine monitoring of water quality, optimised farming practices, and the exploration of natural antimicrobials, are introductory to mitigating the impact of this pathogen [3].

According to microbiological studies, red pitahaya (*Selenicereus costaricensis*) peel methanol extract also showed promising antimicrobial activity, with inhibition zones of 10.18 mm against *L. garvieae* and minimum inhibitory concentrations (MICs) of 80 µg/µL, depending on the preparation [11]. More efficiently, anise (*Pimpinella anisum*) essential oil (EO), with an MIC of 0.312 µL/mL, and a mixed EO of oregano (*Origanum vulgare*) and Echinacea (*Echinacea purpurea*) at MIC ≥ 6. 25 μL/mL exhibited strong antimicrobial activity against *L. garvieae*, performing comparably to some antibiotics in laboratory settings [24,25]. Moreover, the mixed EO demonstrated a greater synergetic efficacy than Florfenicol, a commonly used aquaculture antibiotic. Moreover, dandelion (Taraxacum officinale) root extract (DRE) has displayed promise as a dietary additive for fish, which improved intestinal histology, as evidenced by the increased villi length, villi width, absorption area, goblet cell count, and thickness of the tunica muscularis of rainbow trout [10]. A challenge test revealed that 2.5 mL/kg of DRE in feed significantly enhanced disease resistance against *L. garvieae*, while, for growth optimisation, a concentration of approximately 23.91 mL/kg was suggested for a dual role in health and productivity enhancement [10]. An example of capsicum (*Capsicum annuum*) oleoresin supplementation (0.7% in feed) improved the innate immune response of rainbow trout, denoted by elevated respiratory burst activity, total protein, globulin, and immunoglobulin levels [26]. Histological examinations confirmed no adverse effects at this optimal dose, further supporting its integration into sustainable aquafeed formulations. Lastly, the gene expression analysis showed upregulated pro-inflammatory markers such as IL-1β, IL-8, TGF-β, and IgT, enhancing disease resistance [26]. Likewise, substantial protection from lactococcosis was previously noted following a four-week treatment using a feed additive derived from a multi-citrus extract (Biocitro^®^) at 750 mg/kg of feed, administered at a daily rate of 1.5% of body weight [27].

Other natural antimicrobials, such as cocoa (*Theobroma cacao*) pod husk extract (CPH) and red-seaweed-derived carrageenan (CGN), have demonstrated immunostimulatory effects [28,29]. For instance, CPH enhanced non-specific immune responses, including phenoloxidase activity, phagocytosis, and respiratory bursts, and regulated carbohydrate metabolism, improving resistance in prawns (*M. rosenbergii*) [29]. Similarly, CGN-enriched diets (20 g kg^−1^) enhanced antioxidant enzyme activity and regulated pro-inflammatory cytokines in cobia, with reduced mortality rates in cobia fish under the *L. garvieae* challenge [28]. Valerian (*Valeriana officinalis*) and passionflower (*Passiflora incarnata*) extracts, incorporated into rainbow trout feed, promoted growth, improved haematological indices, and enhanced immunological markers [22]. These phytogenic additives increased the expression of immune-related genes such as lysozyme II and IgM while downregulating stress-related markers like hsp70 [22]. Post-challenge survival rates in treated groups significantly exceeded those of controls, affirming their protective function. Once more, Andiz (*Juniperus Drupacea*) root extract indicated efficiency against *L. garvieae* infection in Archocentrus centrarchus [30]. Administered intraperitoneally at 0.01 mL g⁻¹, the extract greatly enhanced immune and antioxidant responses (glutathione S-transferase and SOD) compared to conventional oxytetracycline and control groups. A real-time PCR analysis showed a 14-fold boost in TNF gene expression and a 5.5-fold upsurge in IL gene expression in extract-treated fish, far surpassing the effects observed in antibiotic-treated groups [30].

Leveraging advancements in natural antimicrobials, the study of phages, vaccines, and peptides presents complementary and novel approaches toward improving disease management and strengthening fish health against *L. garvieae* [9,31,32,33,34,35]. Lytic bacteriophage PLG-II has emerged as an antimicrobial agent against *L. garvieae* [32,33]. PLG-II, isolated from seawater in Japan, exhibited strict lytic activity against serotype II strains of *L. garvieae* without infecting serotype I strains. Genomic analyses revealed the absence of lysogenic and resistance genes, making it a safe candidate for therapeutic applications [32,33]. Challenge experiments showed that fish-fed bacteriophage-supplemented diets achieved a 100% survival rate, demonstrating its potential as a biological control strategy.

Vaccines, particularly bacterial ghost vaccines (BGVs), have demonstrated strong immunogenicity against *L. garvieae* [34]. BGVs prepared using the NaOH-induced lysis of *L. garvieae* strains have shown noteworthy immunostimulatory effects in fish models, enhancing innate and adaptive immune responses. Experimental Nile Tilapia vaccinated with monovalent (LgG) or bivalent (SiLgG) BGVs exhibited elevated levels of immune markers such as IL-1β, SOD, CAT, MHC-II, and CD4, along with a reduction in MDA, and were correlated with high relative survival percentages (RPS), with the bivalent vaccine achieving a special ≈ 95% RPS [34].

Further advances in immunostimulants by dietary supplementation with HK L-137, a heat-killed LAB (*Lactobacillus plantarum* L-137), improved survival rates and upregulated immune-related genes such as IL-12, TNF-α, and IFN-γ in yellowtail (*S. quinqueradiata*) [31]. Previously, aqueous vaccines combined with oil adjuvants significantly increased the expression of immune markers like IgM, TNF-α, and IL-1β in golden pompano, providing strong protective efficacy against *L. garvieae* [9].

Of the latest studies, antimicrobial peptides, such as TroH2A-29, have also shown promise in combating *L. garvieae* [35]. TroH2A-29 exerted its antibacterial effects by disrupting *L. garvieae* cell membranes, leading to a leakage of cellular contents and bacterial death. Transmission electron microscopy confirmed the structural collapse of *L. garvieae* cells treated at the MIC50 of 60 μM, effectively permeabilising cell membranes and inducing membrane depolarisation [35].

The aims of this study were to investigate if a mixture of organic acids (natural antimicrobials-Aq) can in any way interfere with the virulence mechanisms of *L. garvieae* and reduce its pathogenicity in an in vitro infection model using the CHSE-214 cells (fish cell line derived from Oncorhync tshawytscha embryos). The efficacy of Aq against various fish pathogens has been extensively studied [21] and we aimed to extend our knowledge on the biological mechanisms sitting behind its mechanism of action.

## 2. Results

### 2.1. Bacterial MIC, MBC, Growth Curves, EPS Production, and Biofilm Formation in the Presence of Aq

**Figure 1:**
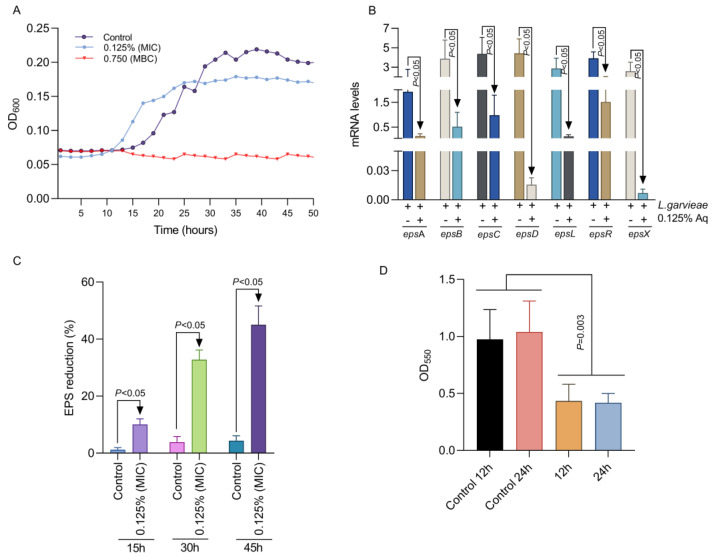
The impact of Aq MIC and MBC concentrations on *L. garvieae* growth profile (**A**), EPS gene expression at 45 h of exposure (**B**), and the EPS production profiles (**C**). Panel (**D**) represents the impact of Aq on *L. garvieae* biofilm formation at 12 and 24 h of exposure to 0.125% Ag. The experiments were repeated three times with the Student *t* test being used to quantify significance.

Our first aim was to establish the MIC and the MBC concentrations at which Aq is most efficient in inhibiting *L. garvieae* growth. Inhibitory activity was detected at 0.125% Aq (MIC) and a minimum bactericidal concentration at 0.75% Aq (MBC). The MIC and MBC concentrations were further used to test their impact on *L. garvieae* growth profiles. As indicated in Figure 1A, at the MIC (0.125%) concentration, Aq starts to inhibit the growth of *L. garvieae*, and whether the MBC concentration (0.75%) bacterial growth succumbs completely. To further investigate the impact on bacterial virulence factors, we have tested the impact on EPS production at the MIC concentration. We have shown that, at 0.125% Aq, the mRNA levels produced for the *eps*A, *eps*B, *eps*C, *eps*D, *eps*L, *eps*R, and *eps*X genes were significantly (*p* < 0.05) reduced (Figure 1B). Moreover, the reduction in mRNA levels was associated with a significant (*p* < 0.05) reduction in EPS detection in the culture supernatants (Figure 1C). The observed reduction in EPS production also caused a significantly reduced (*p* = 0.003) ability of *L. garvieae* to produce biofilm (Figure 1D). The concentration of 0.125% was further used to evaluate the effect of Aq in preventing the *L. garvieae* infection of CHSE-214 fish epithelial cells and further evaluate its anti-pathogenic specificity.

### 2.2. The Impact of Aq on the Ability of L. garvieae to Infect CHSE-214 Cells and Reduce Bacteria-Induced Cytotoxicity

**Figure 2:**
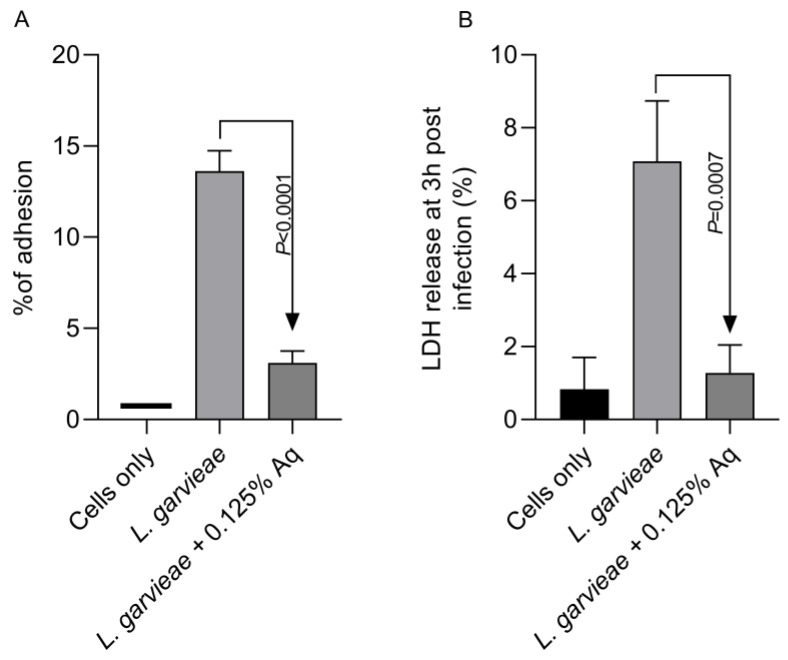
The impact of Aq on the adhesion of *L. garvieae* to CHSE-214 cells (**A**) and on bacteria-induced cytotoxicity (**B**). The *p* value indicates a very significant difference when 0.125% Aq was added to the infection assay (*p* < 0.0001). The experiments were repeated three times, with the Student *t* test being used to quantify significance. The bar charts were calculated and plotted using GraphPad Prism 11.

**Figure 3:**
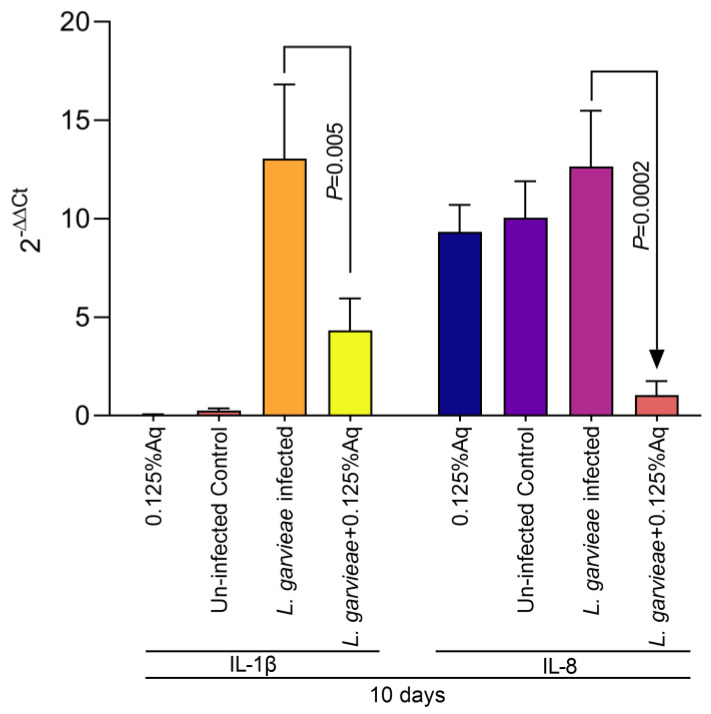
The impact of Aq on the CHSE-214 IL-1β and IL-8 expression after *L. garvieae* infection. Data are expressed as 2^−ΔΔCt^ where Δ^Ct^ = Ct (target gene) − Ct (housekeeping); values are the mean of three test replicates. The experiments were performed in triplicate and on three separate occasions and *p*-values are presented on the graph.

Based on the MIC value, the concentration of 0.125% Aq was further selected to assess its impact on bacterial adhesion to the CHSE-214 cells (Figure 2A). To quantify the number of cell-associated bacteria, infected monolayers were washed three times with PBS and treated with 0.1% Triton X-100 in PBS. Tenfold dilutions of each well were plated and colonies enumerated after 2 days of incubation, as described in Materials and Methods. At the MIC concentration, Aq was able to significantly reduce *L. garvieae* attachment to CHSE-214 cells (*p* < 0.0001) when compared to the infected and untreated cells. The LDH release experiment, at 3 h post-infection, revealed that, in the absence of Aq, *L. garvieae* was significantly more cytotoxic (*p* = 0.0007) than in the presence of 0.125% Aq (Figure 2B). These results clearly indicate that Aq was able to inhibit significantly the ability of *L. garvieae* to adhere to the CHSE-124 cells and to reduce bacteria-induced cytotoxicity. Furthermore, the reduction in *L. garvieae* virulence was also accompanied by a significant reduction in IL-1β (*p* = 0.005) and IL-8 (*p* = 0.0002) proinflammatory cytokine expression, emphasising further the anti-pathogenic effect of Aq (Figure 3).

### 2.3. Ex Vivo Effect of Aq in Preventing L. garvieae-Induced Haemolysis in Fish Red Blood Cells (RBCs)

**Figure 4:**
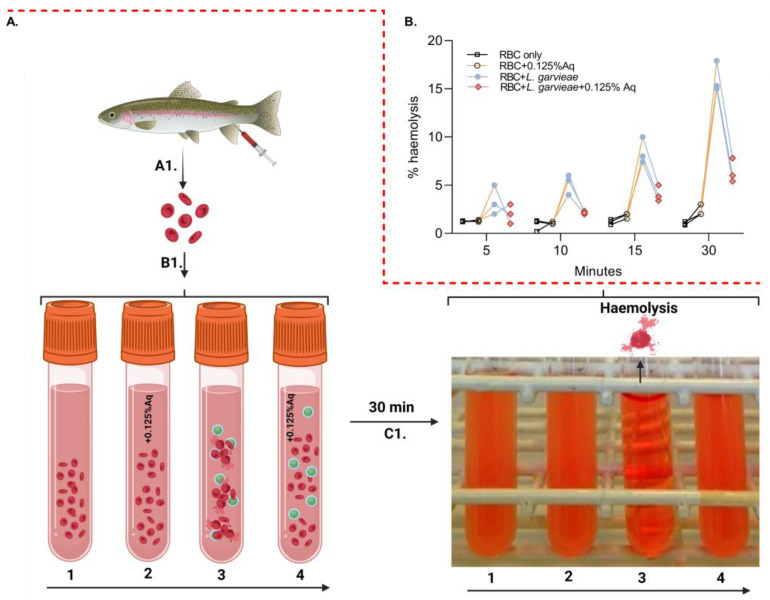
The impact of Aq in preventing *L. garvieae*-induced RBC haemolysis. Panel (**A**) describes the experimental design, which includes panel (**B1**) [RBC-only experiment (1), RBC + 0.125% Aq (2), RBC + *L. garvieae* (3), and RBC + *L. garvieae* + 0.125% Aq (4)]. The impact on RBC haemolysis is presented in panel (**C1**) and the corresponding measurements in panel (**B**) of all experimental combinations after 30 min. The inclusion of 0.125% Aq resulted in a significant reduction in RBC haemolysis (*p* = 0.02). The triplicate experimental data were analysed by one-way ANOVA. Panel (**A**) was designed with Biorender.com.

To further investigate the role of Aq in reducing post-infection pathogenicity consequences, we have designed an ex vivo haemolysis experiment in the presence of *L. garvieae*. The experiment was designed as described in the Material and Methods section and included an RBC-only experiment (1), RBC + 0.125% Aq (2), RBC + *L. garvieae* (3), and RBC + *L. garvieae* + 0.125% Aq (4) (Figure 4A-B1). The results indicate that *L. garvieae* was significantly less able to (*p* = 0.02) cause RBC haemolysis as indicated in Figure 4B. These results showed that the decrease in haemolysis was similar regardless of the time point at which haemolysis was measured through the 30 min duration of the experiment. Conclusively, these results show that Aq is inhibiting the haemolytic activity of *L. garvieae* potentially through the downregulation of outer surface haemolytic regulators or via the reduction in EPS release.

### 2.4. The Gene Expression of hly1, hly2, and hly3 After Exposure in Culture to 0.125% Aq

**Figure 5:**
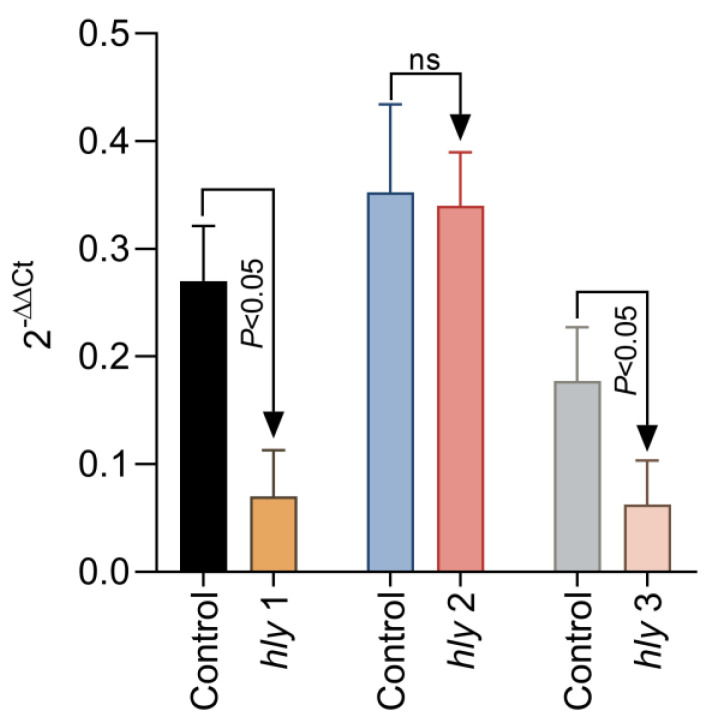
The impact of Aq on the *L. garvieae hly*1, *hly*2, and *hly*3 gene expression. A bacterial culture at OD_600_ of 3 was exposed to 0.125% Aq for 1 h. Data are expressed as 2^−ΔΔCt^ where Δ^Ct^ = Ct (target gene) − Ct (housekeeping); values are the mean of three test replicates. The experiments were performed in triplicate and on three separate occasions. *p*-values of less than 0.05 are indicated on the graph. (ns – not significant).

To further investigate the mechanism of reduced haemolysis, we have next tried to investigate if the reduction in *L. garvieae* haemolysis of fish RBCs reflects a lack of expression in surface haemolysin genes *hly*1, *hly*2, and *hly*3. To achieve this, we have added 0.125% Aq to the *L. garvieae* log phase culture, exposed for a duration of 1 h. Our results show (Figure 5) that a significant downregulation was achieved for *hly*1 and *hly*3 (*p* < 0.05); however, no significant change was detected in the expression of *hly*2 after 1 h of exposure to 0.125% Aq. The control culture received no Aq. These results, coupled with the haemolytic activity on RBCs, clearly indicate that Aq can modify the expression of bacterial surface proteins involved in *L garvieae* haemolytic activity.

## 3. Discussion

Outbreaks of lactococcosis typically occur during spring and summer when water temperatures exceed 16–18 °C [3,4]. Field observations indicate that trout weighing 150–600 g are highly susceptible, although juveniles as small as 10–80 g can also develop symptoms under experimental conditions [4]. *L*. *garvieae* is highly transmissible, spreading horizontally through contaminated water and vertically from infected broodstock to offspring. High-density farming practices exacerbate outbreaks, as the close contact between fish facilitates rapid disease spread. The pathogenesis of *L*. *garvieae* begins with its entry through the primary mucosal sites of the host, including the gills, eyes, and gastrointestinal tract. Once inside, the bacterium colonises and proliferates in tissues such as the gills, spleen, kidney, and liver, spreading systemically via the bloodstream. Early symptoms in infected fish include anorexia, lethargy, and the darkening of the skin (melanosis) [36]. As the infection progresses, more severe signs develop, including exophthalmia (bulging eyes), abdominal swelling, anal prolapse, and haemorrhages on the skin, fins, and internal organs [36]. The accumulation of ascitic fluid in the peritoneal cavity, necrosis in the liver and spleen, and yellowish exudates covering the brain are common pathological findings in severe cases [3,36].

Some evidence has recently emphasised the efficacy of natural antimicrobials, including plant extracts, peptides, essential oils, and immunostimulants, in benefiting fish resistance against *L. garvieae* [11,22,24,25,26,27,28,29]. These agents act mainly via direct antimicrobial properties, particularly improving fish’s immune and physiological parameters, presenting a possible holistic approach to infection resolution. One of the main virulence factors are the bacterial polysaccharides, which play a pivotal role in immune evasion and prevents recognition and phagocytosis by host immune cells by masking surface molecules that would otherwise trigger a defensive response, allowing *L*. *garvieae* to persist in the host and spread systemically, leading to severe infections [36,37,38]. This adhesion is a condition for further tissue damage and systemic invasion, whereas the presence of adhesion clusters (e.g., *adhC* and *adh*) enhances this capacity, promoting robust attachment to host cells and tissues [39]. Furthermore, the generation of haemolysins, including *hly1*, *hly2*, and *hly3*, equips it with a high pathogenicity by mainly inflicting lysis in red and white blood cells [15,38,39,40]. In turn, haemolysins create pores in cell membranes, disrupting cellular integrity and facilitating bacterial spread and tissue invasion [4,40]. In parallel, the production of enzymes such as NADH oxidase and SOD aids in combating oxidative stress during infection, allowing *L*. *garvieae* to survive in hostile host environments [37,39,40]. Adhesion is another critical component of *L*. *garvieae* pathogenicity [15,36,38]. Adhesin proteins, such as adhesins (*adh*), *adh*CI-II, *adh*PavA, *adh*PsaA, and LPxTG (I-III)-containing surface proteins, enable them to adhere to host tissues, starting colonisation and infection [4,15,36,38,39].

*L. garvieae* is responsible for causing fatal haemorrhagic septicaemia in cultured fish species leading to significant economic losses [41]. The Aq-induced reduction in *L. garvieae* EPS and haemolytic capacity links to its virulence abilities and is associated with the formation of bacterial capsule polysaccharides [42] and to the presence of haemolysin bacterial surface proteins (Hly1 and Hly2) [43]. Bacterial exopolysaccharides (EPSs) are polymers with a direct role in influencing bacteria–host interactions [44] and have an important role in cell adhesion, protection against environmental conditions and antimicrobials, ultimately facilitating the formation of biofilm [45]. However, the production of EPS can be reduced; for example, 10-Hydroxy-2-decenoic acid (10-HDA), a component present in royal jelly, can influence the ability of *S. aureus* to produce biofilm and can inhibit its haemolytic activity [46]. This anti-haemolytic activity of natural antimicrobials against *Staphylococcus aureus*-induced haemolysis was also proven for flavonoids [47]. Essential oils (lemon verbena) have been shown to inhibit EPS production in *Pseudomonas* D4, leading to reduced haemolytic activity and biofilm formation levels [48]. *L. garvieae*, also an α-haemolytic bacterium, can cause the lysis of red and white blood cells via cell membrane pore insertion and the destruction of their membrane during infection [49]. As a consequence, this could lead to further lesions of the vascular endothelium or other internal organs [50]. Our experimental data indicate that Aq can prevent red blood lysis when induced by the presence of *L. garvieae* in culture. This anti-haemolytic effect of natural antimicrobials (plant extracts) is manifested by inhibiting haemolysis bacterial surface proteins which are directly targeted [51]. It becomes clear that extracts will not express their anti-haemolytic activity by directly protecting the red blood cells [52], but, rather, through a direct impact on bacterial surface proteins as observed in our study. Moreover, reduced haemolysis will also impact on the meat/muscle quality by preventing haemoglobin oxidation events postmortem [53].

*L. garvieae* infections also lead to a significant increase in IL-8 and IL-1β in the spleen and kidneys of rainbow trout, cytokines now considered immunological markers useful in selective breeding programs [54]. Coincidently, IL-1β, a cytokine known to be involved in prompting defensive cells against the disease [55], is expressed at significantly lower levels in Aq-infected and -treated CHSE-214 cells in our study. Alongside IL-1β, the expression of IL-8, a cytokine involved in the recruitment of monocytes, neutrophils, and lymphocytes and in the activation of phagocytosis [55], was also significantly downregulated. The lack of IL-1β and IL-8 response in our CHSE-214 infected cells is probably caused by the decreased infection levels in the presence of Aq. Cytotoxicity represents another measure of *L. garvieae* virulence abilities and pathogenicity, usually measured through the amount of lactate dehydrogenase (LDH) released by the infected host cells [37]. In our work, the presence of Aq during the infection of CHSE-214 cells resulted in significantly less bacteria attached to the cells and a significant reduction in LDH released by the infected cells. These results clearly indicated Aq can reduce the *L garvieae* attachment to fish epithelial cells, therefore resulting in diminished virulence.

## 4. Materials and Methods

### 4.1. CHSE-214 Cell Line and Organic Acid Blend

The CHSE-214 epithelial cells were obtained from the European Collection of Authenticated Cell Cultures (ECACC). The CHSE-214 cells (ECACC No. 91041114) are a fish cell line derived from *Oncorhync tshawytscha* embryos and previously used in shrimp related studies [56]. Cells were cultured in minimum essential medium (MEM) (ThermoFisher Scientific, Renfrew, UK) supplemented with 10% foetal bovine serum (ThermoFisher Scientific, Renfrew, UK), 2 nM L-glutamine (Corning), and an antibiotic/antimycotic solution (10,000 IU/mL penicillin, 10,000 μg/mL streptomycin, and 25 μg/mL amphotericin B). *Lactococcus garvieae* (*L. garviae*) was grown as necessary in BHI media at 37 °C. The organic acid mixture (AuraAqua) contains lactic acid, and E330 citric acid and citrus extract was used, along with 5% maltodextrin, 1% sodium chloride, 42% citric acid, 18% sodium citrate, 10% silica, 12% malic acid, 9% citrus extract, and 3% olive extract (*w/w*). The raw materials were supplied by Bioscience Nutrition Ireland (Auranta, Ireland).

### 4.2. Minimum Inhibitory Concentration (MIC), Minimum Bactericidal Concentration (MBC), and Infection Assay

The MIC and the MBC of AuraAqua against *Lactococcus garvieae* (*L. garvieae*) was determined using the two-fold tube dilution method. AuraAqua dilutions (8% to 0.015625% *v*/*v*) were performed in Brain Heart Infusion (BHI) broth. Overnight cultures were collected via centrifugation, washed in PBS two times, and re-diluted in BHI broth to 1 × 10^6^ CFU/mL. Each vial was inoculated with 5 × 10^5^ CFU/mL of the bacterium. Separate bijou (5 mL) with growth media, with or without AuraAqua or bacteria, were used as positive controls following growth at 37 °C for 24 h in aerobic conditions. There was an absence of visible growth above the MIC. A volume of 100 mL was taken from each vial, for inoculation, and placed for 24 h at 37 °C on BHI agar plates. Negative controls, including BHI, with or without AuraAqua or bacteria, were also included. The sub-inhibitory concentrations were estimated by exposing the pathogens to different concentrations of the antimicrobial mixture. All experiments were performed in triplicate and on three different occasions. For infection trials, 100 mL of BHI broth was inoculated with 50 μL of the frozen isolate.

### 4.3. Growth Curve

The growth curves of *L. garvieae* was assessed in BHI broth. A volume of 50 µL of the bacterial solution was added to 5 mL BHI in 10 mL sterile tube and incubated at 37 °C and 220 rpm. Optical density was monitored at 600 nm at intervals of 2 h, over 20 h, using an automatic plate reader (FLUOstar Omega, Premier Scientific, Belfast, UK). The antimicrobial concentration that did not inhibit bacterial growth was used for the subsequent phenotypic virulence assays. Each experiment was performed in triplicate.

### 4.4. Infection Assay

The *L. garvieae* cultures were shaken (100 rpm) at 27 °C for 24 h. Absorbance (at 600 nm) of known bacterial densities was determined to obtain a standard calibration curve. An initial bacterial suspension containing 10^7^ CFU/mL was made from the flask broth culture. Subsequent dilutions were made from the above suspension, which were then used in tests. Briefly, monolayers of CHSE-214 cells were prepared in 24-well plates at 1 × 10^6^ cells/well. For infection, *L. garvieae* was grown in BHI media to an OD_600_ of 0.3. A volume of 200 µL of bacterial culture was used to infect the CHSE-214 cells, and cells were infected for another 3 h. The infected monolayers were washed with ice-cold 0.1% Saponin in PBS (3×) for 15 min at 16 °C to permeabilise the cells. Cells were diluted in PBS (1×) collected by centrifugation (6000× *g*) for 10 min at 4 °C. The cell lysates and treated bacterial supernatant of the infected group and bacterial supernatant of the control group were diluted using a 10-fold serial method, cultured on BHI, incubated at 37 °C overnight, and CFU counts were determined.

### 4.5. Anti-Inflammatory Activity in L. garvieae Infected CHSE-214 in the Presence of 0.5% Aq

The expression of IL-8 and IL-1β was measured as previously described [19]. The 2^−ΔΔCt^ method was used to analyse the relative expression (fold changes), calculated relative to the control group. To estimate the gene expression, untreated cells (control) were set as value 1 and treated cells values were compared to this value. Each experiment was repeated 3 times and at 3 separate occasions. Stimulated sample was compared to its control at each time point. *p* values ≤ 0.05 were considered significant.

### 4.6. Cytotoxic Lactate Dehydrogenase (LDH) Release Assay

LDH release was used to identify the impact of Aq on cytotoxicity of *L. garvieae* upon infection of CHSE-214 cells. Measurements were performed as previously described [37]. Briefly, after 3 h of infection, LDH was measured in the infection supernatants. LDH was measured using a cytotoxicity detection kit (Roche, Buckinghamshire, UK), strictly following the manufacturer’s instructions, and detected using spectrophotometric readings at 500 nm (FLUOstar Omega, Premier Scientific, Belfast, UK). Each experiment was performed in triplicate.

### 4.7. Exopolysaccharide (EPS) Content Determination, Haemolysin, and Gene Expression

To further investigate and measure the EPS production, supernatant produced by *L. garvieae* grown in BHI broth (15, 30, and 45 h), in presence or absence of 0.125% Aq, was centrifuged and filtered through a 0.22 µm filter membrane, as previously described [57]. Upon filtration, three volumes of chilled 100% ethanol was added and incubated overnight at 2 °C to precipitate the EPS. The ethanol wash was removed by repeated centrifugation steps and the EPS was quantified using the colorimetric phenol-sulphuric acid method. The bacterial pellet was used further to characterise the impact of 0.125 Aq on the exopolysaccharide gene expression profiles *eps*A, *eps*B, *eps*C, *eps*D, *eps*L, *eps*R, *eps*X, and the control gene for 30S rRNA [37]. RNA was isolated using RNeasy Plus Mini Kit (Qiagen, Manchester, UK). The RNA was reverse-transcribed using Transcriptor First Strand cDNA Synthesis Kit (Roche, Dublin, Ireland) according to the manufacturer’s protocol. The mRNA levels were determined by quantitative RT-PCR using QuantiNovaSYBR Green PCR Kit (Qiagen, Manchester, UK) on a LightCycler 96 (Roche). Primers are presented in Table 1. The gene expression profiles were obtained from 10 separate samples for each gene individually at 45 h growth. For *hly*1, *hly*2, and *hly*3 gene expression, the primers are also included in Table 1 and the RT-PCR was performed as described above with the expression being investigated after exposure of *L. garvieae*, in culture (0.3 OD), to 0.125% Aq for 1 h.

### 4.8. Haemolysis Assay

Preparation and harvesting of trout fish red blood cells (RBCs) were performed as previously described, including the quantification of haemolysis [47]. Briefly, *L. garvieae* cells were diluted at 1:100 in BHI medium and cultured in the presence of RBCs with or without 0.125% Aq for 30 min. The RBCs were prepared as previously described from blood isolated as previously described [53] and added to the lysis assay after separation by centrifugation at 1000× *g* for 5 min. The experimental protocol was designed to include an RBC-only experiment (1), RBC + 0.125% Aq (2), RBC + *L. garvieae* (3), and RBC + *L. garvieae* + 0.125% Aq (4). The haemolytic activity was measured in the collected supernatants following centrifugation at 16.600× *g* for 10 min and measured using the automatic plate reader (FLUOstar Omega, Premier Scientific, Belfast, UK) at OD_543_. PBS buffer only was used as control without Aq or *L. garvieae*. The study was performed according to the PREPARE and ARRIVE guidelines. All animal procedures were approved by the University of Life Sciences King Mihai I of Romania from Timisoara with the ethical decision 481/2024, according to the European Union’s Directive (2010)/63/EU for animal experimentation and the Romanian Law 205/2004 (art. 7, 8, and 22).

### 4.9. Biofilm Formation in 6-Well Plates

*L. garvieae* agar-grown colonies were used to inoculate 10 mL of BHI media, and the strain was incubated overnight at 37 °C. After 24 h, the overnight culture was diluted 1:100, and washed twice with BHI, followed by centrifugation for 10 min at 7000 rpm. To determine the impact of Aq on *L. garvieae* biofilm formation, the concentration of 0.125% was used. Media, including antimicrobials, were added to each 6-well plate in a volume of 2 mL/well. To avoid evaporation of the inoculum from the wells, each plate was covered with an adhesive seal (Thermo Scientific, Waltham, MA, USA) and further incubated at 37 °C for 24 h. The supernatant was removed by gently washing the plates twice with 2 mL of phosphate-buffered saline (PBS) and air-dried, followed by 2 mL of methanol, which was added to each well to fix the adherent bacteria. After 2 min, the methanol was removed, and the plates were washed twice with sterile PBS and air-dried. The next step was crystal violet (CV) staining, in which 2.25 mL of 0.1% CV solution was added to all wells. After 10 min of dyeing, CV was removed, and the wells were washed twice with PBS and dried. In the last step, 2 mL of 30% glacial acetic acid was added to each well and incubated for 10 min. The absorbance was measured using a microplate reader (FluoStar Omega, Premier Scientific, Belfast, UK) at an absorbance of 550 nm. The assay was repeated thrice on three separate occasions.

### 4.10. Statistical Analysis

Statistical analyses were performed using GraphPad Prism 11 software. Data were represented as mean ± SD. *p*-values <  0.05 were considered statistically significant, following estimations using Student’s *t* test.

## 5. Conclusions

**Figure 6:**
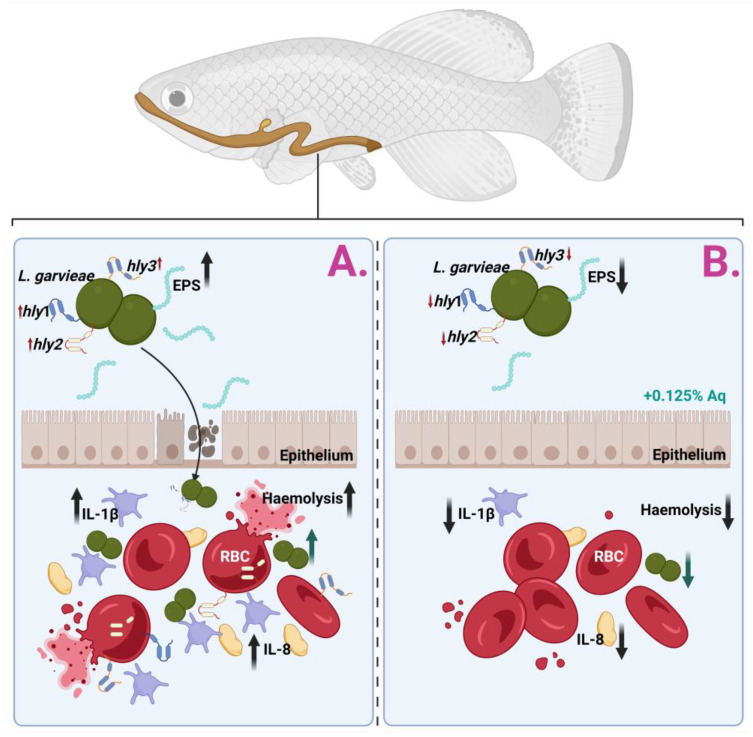
Proposed mechanism of action against *L. garvieae* in an in vitro infection model using CHSE-214 cells.

Resistance to antimicrobial agents adds another complexity to its virulence. Different strains isolated from fish spp. have previously demonstrated antibiotic resistance (i.e., tiamulin, erythromycin, and lincomycin), which can aggravate treatment options and contribute to the pathogen’s persistence in other environments [33]. Contemporary evidence has stressed the role of additional virulence-associated genes, including those encoding enolase (eno), phosphoglucomutase, and exopolysaccharides, which are supplementary and involved in cellular adhesion, metabolic processes, and structural integrity, further enhancing *L*. *garvieae’s* ability to thrive in diverse hosts and environments [37,38,39]. As described in Figure 6, which describes the biological events involved in the *S. garvieae* infection of CHSE-214 cells and the anti-haemolytic effect, the mechanism of action has, as a starting point, the prevention of bacterial adhesion to the epithelium. In the absence of Aq, *L. garvieae* will increase its EPS production in the presence of CHSE-214 cells, pass the epithelial barrier, increase the expression of host proinflammatory cytokines (IL-1β and IL-8), and use the surface proteins *hly* 1, *hly* 2, and *hly* 3 to induce RBC lysis (Panel (A)). Our data clearly show (Panel (B)) that the inclusion of 0.125% Aq during the infection assay will prevent bacterial adherence to the CHSE-214 cells and prevent the proinflammatory response. Moreover, we have observed a significant downregulation of *hly*1, *hly*2, and *hly*3 proteins in the presence of Aq, therefore reducing the bacterium’s ability to cause RBC haemolysis.

## Figures and Tables

**Table 1:** Exopolysaccharide gene expression primers and haemolysin primers.

*eps*A	F-TTATAGCCTCCCCAGTTTACAC R-TTTAGCAGTCTCGTCTGCAATC
*eps*B	F-CGCAAGTGCTAATCTAGCTG R-AGAGAGGCGGAGTATCAATC
*eps*C	F-TAACAACTATCACTGCGACTCC R-TCAGGGTTCTCAATGATTCCAC
*eps*D	F-TTTCTTATTGCGGCTGCATTGC R-CTCATCAATTGAGTGTCGTCTG
*eps*L	F-ACCAATCGTACAGATCAACG R-CTTGAGCCACCACTATCAAG
*eps*R	F-TTTTACCACCGGCTAAAGGAAC R-TTGCAGAACTGTCATTAGGCTC
*eps*X	F-TATTGAAGCAACAGCCTCACTG R-TTTTTGTCTGGGTAACTAGCCC
30S rRNA	F-TACGAACACCGTATCCTTGAC R-TTGTGTTGGTTCGATGATGTCG
*hly*1	F-TCCTCCGACTAGGAACCAAA R-GCCAGCTTCTCGTGCTTATC
*hly*2	F-GAGCAAAAAGCGAGTGAAGG R-GCATCTGGAGCATCAAGTCA
*hly*3	F-CGTGGAGTTATGGCTGGTTT R-CTTGTGGATCTTCGGGTCTT

## Data Availability

N/A
